# Effect of fluid dynamics on decellularization efficacy and mechanical properties of blood vessels

**DOI:** 10.1371/journal.pone.0220743

**Published:** 2019-08-05

**Authors:** Robin Simsa, Xavier Monforte Vila, Elias Salzer, Andreas Teuschl, Lachmi Jenndahl, Niklas Bergh, Per Fogelstrand

**Affiliations:** 1 VERIGRAFT AB, Gothenburg, Sweden; 2 Department of Molecular and Clinical Medicine, Wallenberg Laboratory, Sahlgrenska Academy at the University of Gothenburg, Gothenburg, Sweden; 3 Austrian Cluster for Tissue Regeneration, Vienna, Austria; 4 Department Life Science Engineering, University of Applied Sciences Technikum Wien, Vienna, Austria; Michigan Technological University, UNITED STATES

## Abstract

Decellularization of blood vessels is a promising approach to generate native biomaterials for replacement of diseased vessels. The decellularization process affects the mechanical properties of the vascular graft and thus can have a negative impact for *in vivo* functionality. The aim of this study was to determine how detergents under different fluid dynamics affects decellularization efficacy and mechanical properties of the vascular graft. We applied a protocol utilizing 1% TritonX, 1% Tributyl phosphate (TnBP) and DNase on porcine vena cava. The detergents were applied to the vessels under different conditions; static, agitation and perfusion with 3 different perfusion rates (25, 100 and 400 mL/min). The decellularized grafts were analyzed with histological, immunohistochemical and mechanical tests. We found that decellularization efficacy was equal in all groups, however the luminal ultrastructure of the static group showed remnant cell debris and the 400 mL/min perfusion group showed local damage and tearing of the luminal surface. The mechanical stiffness and maximum tensile strength were not influenced by the detergent application method. In conclusion, our results indicate that agitation or low-velocity perfusion with detergents are preferable methods for blood vessel decellularization.

## Introduction

Peripheral vascular diseases, such as critical limp ischemia, often require replacement of diseased blood vessels with a suitable biomaterial in a process called vascular grafting. Currently, vascular grafting is performed either by utilizing autogenous vessels, such as the saphenous vein or synthetic conduits such as Dacron or PTFE as grafts. However, both techniques have limitations such as potential unavailability of suitable autogenous vessels or thrombogenicity and insufficient long-term patency of synthetic conduits, especially in case of small diameter vessel replacements [1,2]. These limitations have initiated the development of alternative biomaterials for vascular grafting. A promising alternative are decellularized native blood vessels [3].

Decellularization (DC) is the process of removing cells from the extracellular matrix (ECM) of tissues, by physical, chemical and/or enzymatical methods [4–6]. The DC process can alter the properties of the ECM, and therefore a balance between complete removal of cells and preserved integrity of the ECM is an important criterion.

Successful DC of blood vessels, characterized by low DNA content and absence of nuclei in histological analysis [7], has been reported in multiple previous studies [8–11]. Reported DC methods utilize different detergents in various concentrations, as well as diverse methods to apply the detergents onto the native tissue [12]. Previous protocols for tissue DC have used immersion [8,13,14] (static DC), agitation [15–17] (agitation DC) and vessel perfusion [11,18,19] (perfusion DC) with detergents. Static DC has the advantage of being a simple and easily executed process. However, detergents may not affect all parts of the tissue homogenously. Agitation DC has a slightly more complex setup than static DC and achieves a more homogeneous detergent exposure. Whole tissue perfusion allows the most efficient application of detergents, with a homogenous detergent exposure from both the outside and the luminal side of the vessel tissue. However, it also adds shear forces to the process that may inflict tissue damage, and it requires a more complex setup for flow control.

The administration (static, agitation, perfusion) of DC detergents may not only affect the efficacy of cell removal, but also the properties of the decellularized ECM. Parameters such as the stiffness of the ECM are important for the compliance to the native vessel when used as bypass graft, and it influences surface protein signaling, cell proliferation and cell attachment [20–23]. Hence, knowledge about the effect of detergent application on these properties is of vital importance for DC optimization.

In the present study we evaluated how the fluid application of detergents (static, agitation or perfusion setup) on porcine vena cava affects DC efficacy, ECM integrity and mechanical properties of the treated vessels. A previously optimized DC protocol [11], based on the detergents TritonX-100 (TX), Tributyl phosphate (TnBP), and the enzyme deoxyribonuclease (DNase) was used for the DC of vessels.

## Materials and methods

### Animal material

Porcine vena cavas were collected from a local slaughterhouse in Gothenburg, Sweden. The vessels were dissected free from surrounding excess tissue, and was placed in PBS containing 0.5% antibiotic-antimycotic (AA) (Thermofisher, USA), before transported to the lab. The vessels were measured for average length (59.34 mm ± 6.25) and inner diameter (13.11 mm ± 0.96), and was frozen down in PBS containing 0.5% AA at -80°C.

### Decellularization

**Fig 1:**
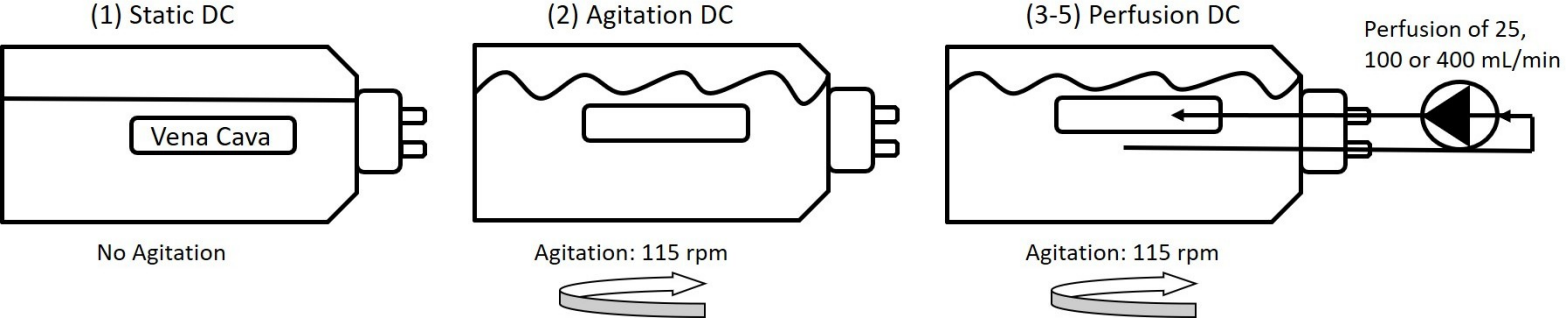
Schematic illustration of the decellularization setup. Porcine vena cavas were decellularized with detergents (1) under static conditions, (2) by agitation at 115 rpm or (3–5) by perfusion, with agitation at 115 rpm and perfusion at 25, 100 or 400 mL/min.

DC of samples was performed at 37°C in standard 250 mL flasks (Duran) either under static conditions, with agitation or with perfusion. In the perfusion setup, the vessels were connected through a tubing at one end to a peristaltic pump (030.3134.3DE, Watson Marlow, USA) as previously described [11] (Fig 1). Five different groups were tested: (1) static DC, (2) agitation DC with agitation at 115 rpm on an incubation shaker (KS 4000i control, IKA, USA), or perfusion DC with agitation at 115 rpm and perfusion of either (3) 25 mL/min, (4) 100 mL/min or (5) 400 mL/min. For perfusion DC, vessels were tied with sutures (Ethicon, Sweden) to a male luer lock with 8.7mm inner diameter (Cole Parmer, USA) in order to connect to tubings with 3.2mm inner diameter (Nalgene Silicone Tubing, VWR, USA). A previously optimized method was applied, utilizing the detergents TritonX-100 (TX) (MerckMillipore, USA) and Tributyl phosphate (TnBP) (MerckMillipore, USA), both at a concentration of 1%, and the enzyme deoxyribonuclease (DNase) (VWR, USA) at a concentration of 40 U/mL [11]. Between each step, vessels were washed 3 times with H2O for 5 minutes. The DC process was initiated with TX for 15h, followed by TnBP for 4h and DNase overnight. Vessels were then incubated again in TX for 8h and in DNase overnight. Finally, vessels were washed for 24h in PBS. Following DC, biopsies for DNA quantification and histology were taken from the edge of the vessel and rest of the vessel frozen in PBS at -80°C for further analysis. As a non-DC control, untreated blood vessels (frozen directly after harvest and not treated with detergents) were used.

### DNA quantification

DNA from 10–30 mg wet tissue (n = 5) was extracted with the DNeasy Blood and Tissue Kit (Qiagen, Germany), followed by quantification with the Qubit ds DNA HS Assay kit (Life technologies, USA), both according to the manufacturer’s protocols. Briefly, tissue pieces were incubated at 55°C while shaking in proteinase K solution until tissue was completely digested. DNA was extracted with a spin column and measured spectrometrically with a Qubit 3.0 Fluorometer (ThermoFisher, USA). The total amount of DNA in ng per mg wet tissue weight was calculated from the supplied DNA standard.

### Histology

Whole mount *en face* preparations and cross sections of paraffin embedded tissues (5 μm thickness) were stained with 4',6-diamidino-2-phenylindole (DAPI) (ThermoFisher, USA). In both cases, 25 μg/mL DAPI in PBS was applied on the sample for 2 minutes in the dark. The samples were analyzed under a fluorescence microscope (Axiovert 40 CFL, Zeiss). Additionally, cross sections were stained with Hematoxylin-Eosin (H&E), following standard procedures.

### Immunohistochemistry

For immunohistochemistry, cross sections of paraffin embedded tissues were treated for antigen retrieval by incubating 15 minutes at 98°C water bath in a Tris-EDTA buffer containing 0.5% Tween (Sigma Aldrich, USA), followed by blocking in 5% FBS for 30 minutes. Samples were stained with antibodies against fibronectin (1:100, ab6328, Abcam, UK), vitronectin (7.5 μg/mL, ab13413, Abcam, UK), elastin (1:100, NB100-2076, Novus Biologicals, USA) and collagen type I/III (1:100, 2150–2555, Bio-Rad, USA) overnight at 4°C in the dark. Secondary antibody incubation was performed with donkey anti-mouse IgG antibodies conjugated to AlexaFluor488 (1:200, A-21202, ThermoFisher, USA) or donkey anti-rabbit IgG antibodies conjugated to AlexaFluor594 (1:200, A-21207, ThermoFisher, USA) for 1h at room temperature. Samples incubated with only secondary antibodies were used as negative control.

### Fluorescence intensity quantification

Fluorescence intensity of samples stained with fibronectin and vitronectin was compared to observe differences in protein abundancy, as previously described [11,24]. Briefly, images of samples (n = 5) were taken at identical microscopy settings regarding magnification, exposure and gain. Then, images were opened in ImageJ (v1.51, NIH) and transformed into 16-bit images. 2 regions of interest on the ECM (with identical area size for all samples) as well as on the background were selected and measured for area, mean and integrated density. This was performed on multiple images of a single sample and results averaged. Fluorescence intensity was then calculated with following formula:

Fluorescence Intensity = Integrated density of sample–(area of sample * mean of background)

The fluorescence intensity was normalized to the native group (100%).

### ECM protein quantification

The amount of the ECM proteins elastin, soluble/insoluble collagen and sulfated glycosaminoglycans (GAGs) was quantified with commercially available kits (n = 5 for all assays). Elastin was quantified with the Fastin Elastin Assay (Biocolor, UK) by extracting elastin from 20–30 mg wet ECM at 100°C in 750 μL 0.25M oxalic acid. This procedure was repeated twice, and extracts pooled. Then, 100 μ of extract were mixed with 100 μL precipitation reagent and supplied protocol followed, after which absorbance was measured at 513 nm. Soluble collagen was quantified with the Sircol Soluble Collagen Assay (Biocolor, UK), by extracting soluble collagen from 20–30 mg wet ECM at RT in 1 mL 0.1M HCl containing 0.1 mg/mL pepsin for 2 days. Then, 50 μL extract were used for the assay, following the suppliers’ protocol, after which absorbance was measured at 555 nm. Insoluble collagen was quantified with the Sircol Insoluble Collagen Assay (Biocolor, UK). Briefly, 15–20 mg wet ECM was incubated at 65°C for 3 h with supplied fragmentation reagent at a ratio of 50 μL reagent per mg ECM. Then, 50 μL extract were used for the assay, following the suppliers’ protocol, after which absorbance was read at 550 nm. Final results were obtained by multiplying with a correlation factor of 2.2, as denatured collagen obtained with this extraction method has 45% lower dye-binding affinity compared to native collagen. The amount of sulfated glycosaminoglycans (GAGs) in the ECM was quantified with the Blyscan Sulfated GAG Kit (Biocolor, UK), by extracting GAGs from 15–30 mg of wet tissue by incubation with 0.1 mg/mL papain (ThermoFisher, USA) in a sodium phosphate buffer at 65°C. When samples were completely digested, 50 μL and the samples were used for the assay, following the suppliers’ protocol, after which absorbance was measured at 656 nm. All assays were also performed on blanks and respective protein standard to allow quantification of protein content.

### Scanning electron microscopy

For scanning electron microscopy (SEM) analysis, 2 individual specimen from 2 samples per group were taken with a biopsy punch (4 mm diameter) and fixed in 2.5% Glutaraldehyde in PBS overnight. Samples were then dehydrated in a series of alcohol concentrations for 15 minutes each (50%, 60%, 70%, 80%, 90% and 100%) followed by critical point drying and sputter-coating with gold (Q150R, Rotary pumped coater, Quorum, UK). SEM pictures were taken with a JEOL JSM-6510 microscope (Jeol GmbH, Germany).

### Cytotoxicity

To test cytotoxicity of vessels with a relevant cell type, human umbilical vein endothelial cells (HUVECs, ThermoFisher, USA) were seeded at a concentration of 2*10^3^ cells/well in endothelial cell growth media (211–500, Sigma, USA) in a 96-well plate. After 2 days of incubation, DC vessel pieces of 5mm diameter were cut out with tissue biopsy puncher and added to wells containing preseeded cells (n = 5) in duplicates. Incubation was continued for 48h, after which DC vessel pieces were removed and MTS reagent (CellTiter 96 AQueous One Solution Cell Proliferation Assay, Promega, Sweden), diluted 1 to 6 in media, was added to cells. MTS reagent is reduced by viable cells into a formazan product and can thus be applied to quantify cell viability. Following incubation for 2.5h at 37°C, absorbance was measured at 490nm and cell number calculated from cells seeded in known density. Control consisted of seeded cells with no ECM pieces added.

### Mechanical tests

Mechanical testing of blood vessels was performed with a Zwick Roell machine as previously described [8]. Vessels were cut into equally sized ringlets (width of 5.5 mm), the thickness and length were measured, and samples clamped on 2 U-shaped metal holders into the testing machine (n = 5 with 3 technical replicates each). Testing was performed with at a constant strain rate of 20 mm/min and the stress-strain curve was recorded. Young´s modulus (also known as Emod) was calculated from the linear phase of stress-strain curve. Maximum tensile strength (F_max_) was also obtained from analysis, and theoretical burst pressure was calculated from Barlow´s formula [8].

### Statistical analysis

GraphPad Prism 7 (GraphPad software Inc., San Diego, USA) was used for statistical calculations and graphs. Statistical significance was calculated by one-way ANOVA, with significance defined as p-value lower than 0.05. The asterisks (*) in graph legends indicates p-values of ≤ 0.05 (*), ≤ 0.01 (**), ≤ 0.001 (***) and ≤ 0.0001 (****). Data is presented as mean ± standard error of the mean.

## Results

### Decellularization efficacy is similar between different flow applications

**Fig 2:**
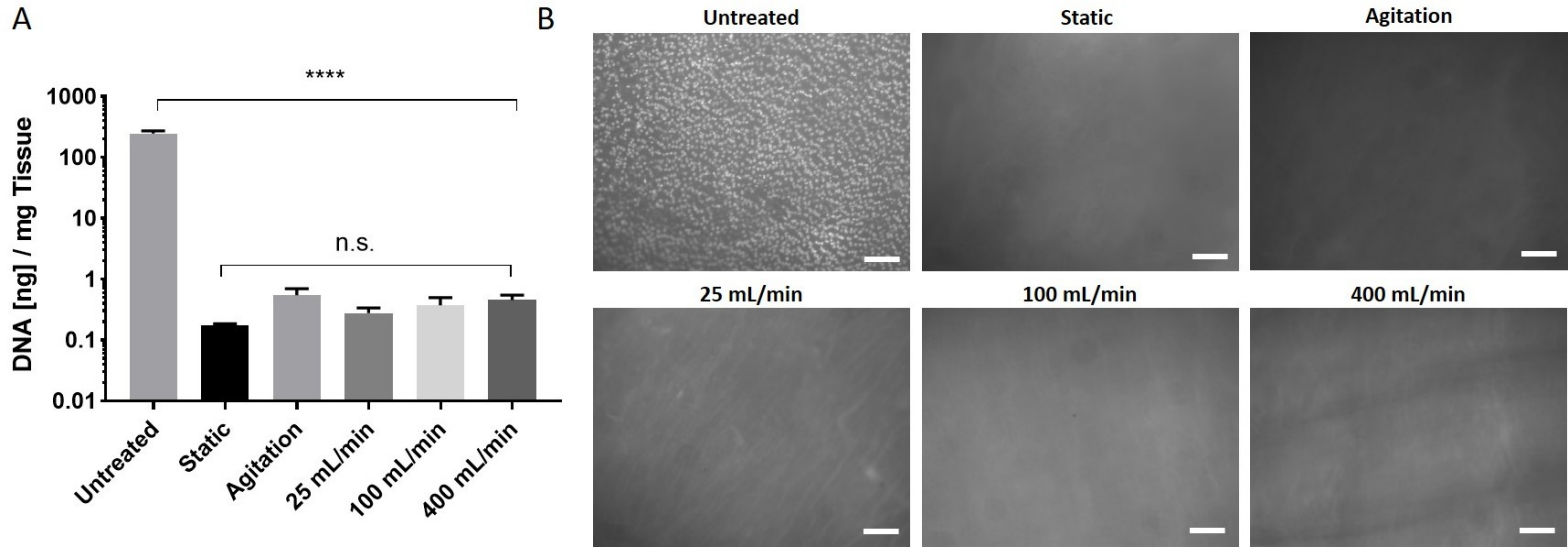
Analysis of remaining DNA content following decellularization. Porcine vena cavas were decellularized with an established protocol utilizing TX, TnBP and DNase. (A) Remaining DNA content in the tissue was measured. Result are shown as DNA in ng per mg wet tissue (n = 5 for all groups). Y-axis uses a logarithmic scale. (B) Whole-mount tissue sections were stained with DAPI and nuclei were visualized under a fluorescence microscope. Scale bar = 100 μm.

**Fig 3:**
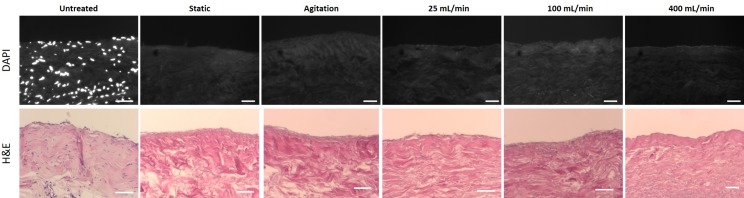
DAPI and H&E of decellularized vessels. Porcine vena cavas were decellularized with TX, TnBP and DNase. Cross sections of paraffin-embedded samples were stained with DAPI (upper panels, nuclei are white) and H&E (lower panels, nuclei are blue). Scale bar = 50 μm.

Porcine vena cavas were decellularized following an established protocol utilizing the detergents TX and TnBP and DNase treatment in different flow application settings. Vessels were incubated with the reagents either under static conditions, under agitation, or with perfusion at 25, 100 or 400 mL/min (Fig 1). Remaining DNA content was used as a measure for decellularization efficacy. We found that all groups had markedly reduced DNA content compared to untreated vessels (p<0.0001), and there was no significant difference between the DC groups (p>0.9999) (Fig 2A). All groups possessed less than 1 ng of DNA per mg of tissue, which translates to less than 1% remaining DNA compared to the untreated tissue. Histological analysis of whole-mount DAPI staining (Fig 2B) and cross-sections stained with DAPI or H&E (Fig 3) confirmed this notion, as no remaining nuclei were found. These results demonstrate that decellularization of porcine vena cava can be achieved by static, agitation, or perfusion-based incubation with DC detergents with no detectable difference in cell removal.

### Insoluble collagen, elastin, fibronectin, and vitronectin are retained following DC, while soluble collagen and GAGs are reduced

**Fig 4:**
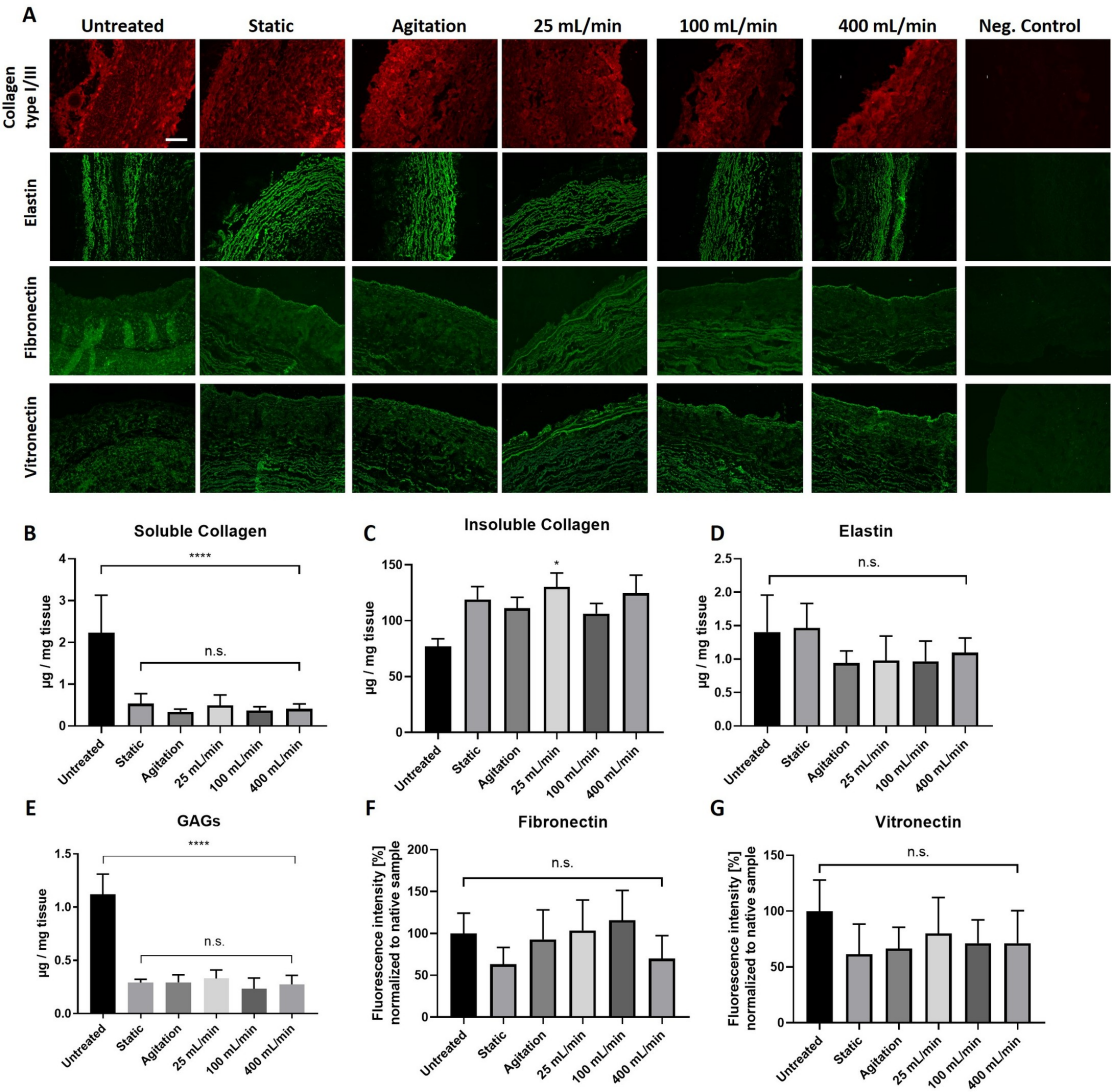
Presence of ECM proteins collagen, elastin, fibronectin, vitronectin and GAGs following decellurarization. Porcine vena cavas were decellularized and analyzed for ECM proteins. (A) Cross-section of paraffin embedded samples were immunostained for collagen type I/III, elastin, fibronectin and vitronectin. Images were taken at identical microscopy settings. Scale bar is 100 μm. (B-E) Quantification of ECM components with commercially available kits from 20–30 mg wet tissue (B) soluble collagen, (C) insoluble collagen, (D) elastin, and (E) GAGs. Fluorescence intensity of samples immunostained for (F) fibronectin and (G) vitronectin. The fluoresvence intensity was quantified with ImageJ, and the values were normalized to the untreated group. n = 5 for all groups.

To analyze presence of common ECM components following decellularization, tissue sections were immunostained against collagen type I/III, elastin, fibronectin, and vitronectin (Fig 4A). Also, soluble collagen, insoluble collagen, elastin, and GAGs were quantified with commercially available kits (Fig 4B–4E). From samples stained with fibronectin and vitronectin, fluorescence intensity was calculated to compare protein abundancy between groups (Fig 4F and 4G). Immunostaining revealed that collagen type I/III, elastin, fibronectin and vitronectin, were present after DC at comparable levels to the untreated group (Fig 4A). This was also shown by protein quantification of insoluble collagen (Fig 4C), elastin (Fig 4D), fibronectin (Fig 4F) and vitronectin (Fig 4G). However, the content of soluble collagen (Fig 4B) and GAGs (Fig 4E) was reduced in the DC groups compared to the untreated group. Soluble collagen however was present at much smaller amounts compared to insoluble collagen in all samples. Thus, in all DC groups the GAGs and soluble collagen content was reduced, while the content of insoluble collagen, elastin, fibronectin, and vitronectin remained unchanged.

### The luminal surface structure of decellularized vessels shows local raptures at higher perfusion velocities

**Fig 5:**
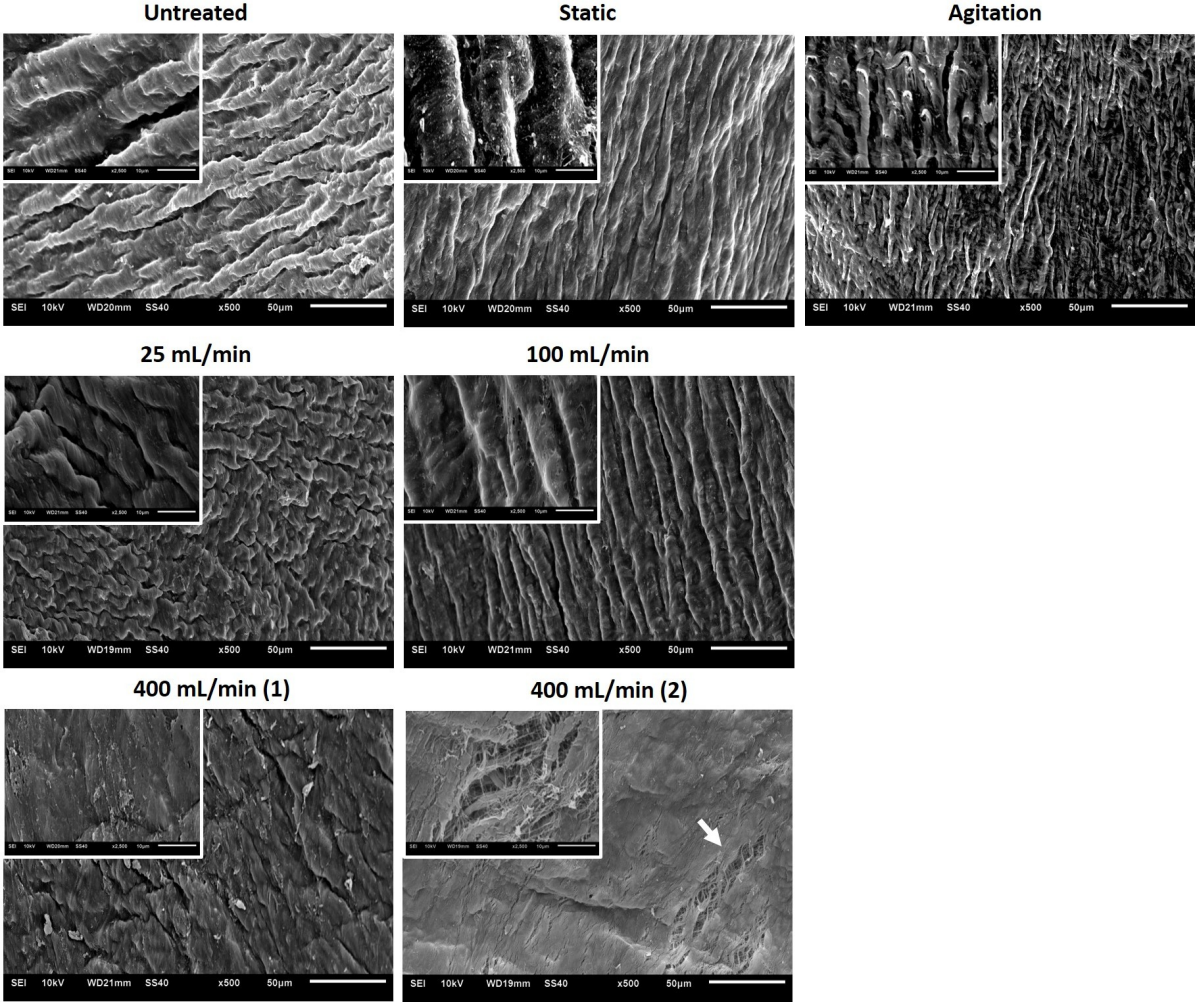
Scanning electron microscopy analysis of porcine vena cava following decellularization. Porcine vena cavas were decellularized and biopsies with a 4 mm diameter were punched out and prepared for SEM analyses. 500x magnification (scale bar = 50 μm), insets show 2500x magnification (scale bar = 10 μm). Two images (from two different samples) for 400 mL/min group show local tearing and flattening of surface to various degrees at different sample areas.

The intraluminal surface structure of decellularized vessels was analyzed with SEM. All groups except the high perfusion group (400 mL/min) had a groovy structure similar to the native untreated vessels (Fig 5). The high perfusion group had a more flattened surface with signs of tearing at some areas (Fig 5). Furthermore, the static DC group had remnant debris on the luminal surface, indicating residual cellular and protein components not removed due to static conditions. These remnants were absent in the other DC groups. In conclusion, agitation or low-perfusion velocities did not seem to negatively affect the ECM surface structure, while the 400 mL/min perfusion the static conditions showed signs of tearing and remnant debris respectively.

### None of the ECM samples show cytotoxicity to cultured HUVECs

**Fig 6:**
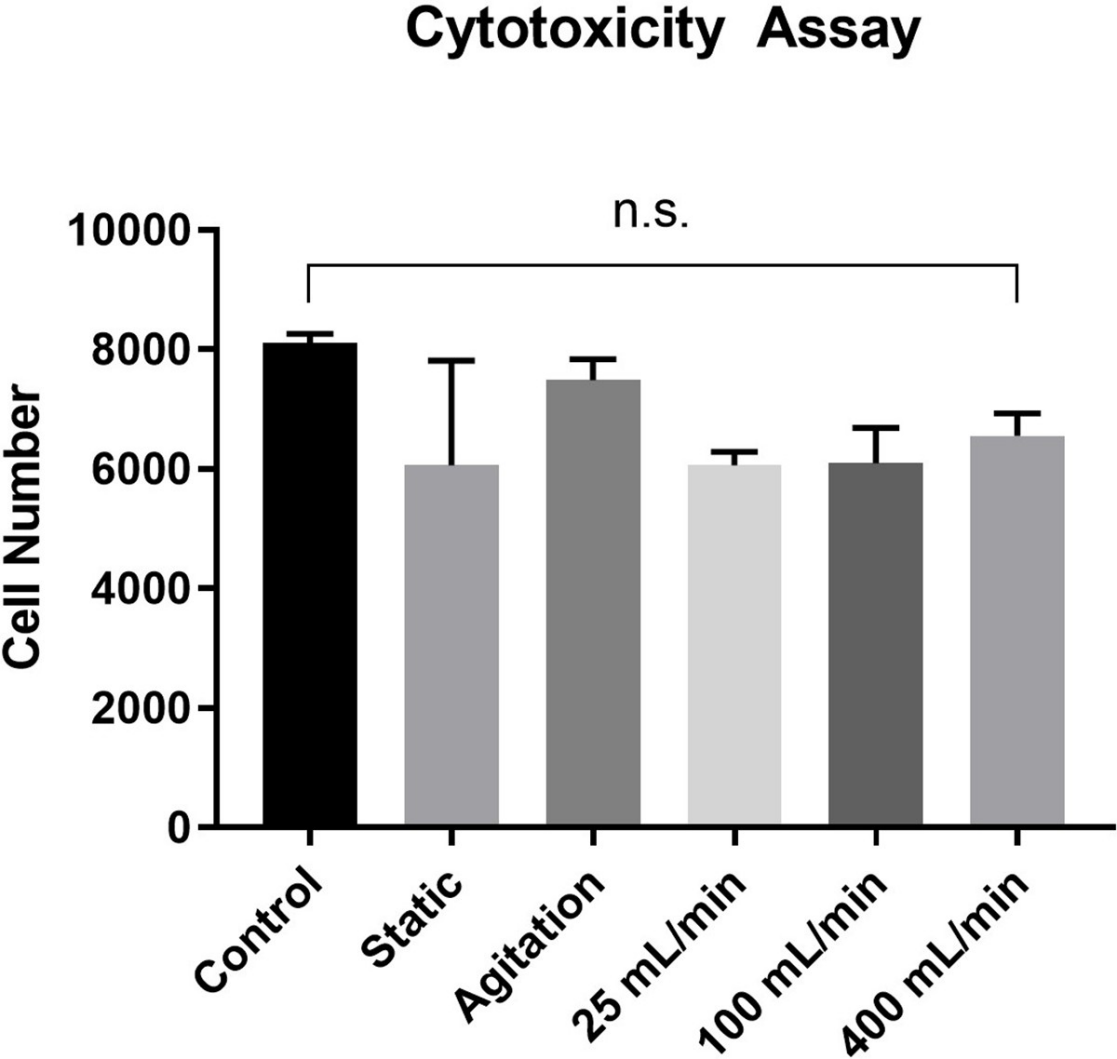
Cytotoxicity of ECM samples. Equally sized ECM pieces were added to HUVECs seeded in a 96-well plate and cell number was measured with MTS assay 48h after incubation. Control consists of cells without added ECM pieces. n = 5 in duplicates for all groups.

To assess whether detergents were sufficiently removed by the washing steps in all DC groups, HUVECs were incubated with or without ECM pieces and number was measured after 48h of incubation. No difference in cell number was found between DC groups and control (cells without added ECM, p = 0.49, Fig 6). This demonstrates that the level of cytotoxic components, such as remnant detergents, was low in all groups.

### Mechanical strength and stiffness remain intact in all DC groups, but wall thickness decreases with higher perfusion velocities

**Fig 7:**
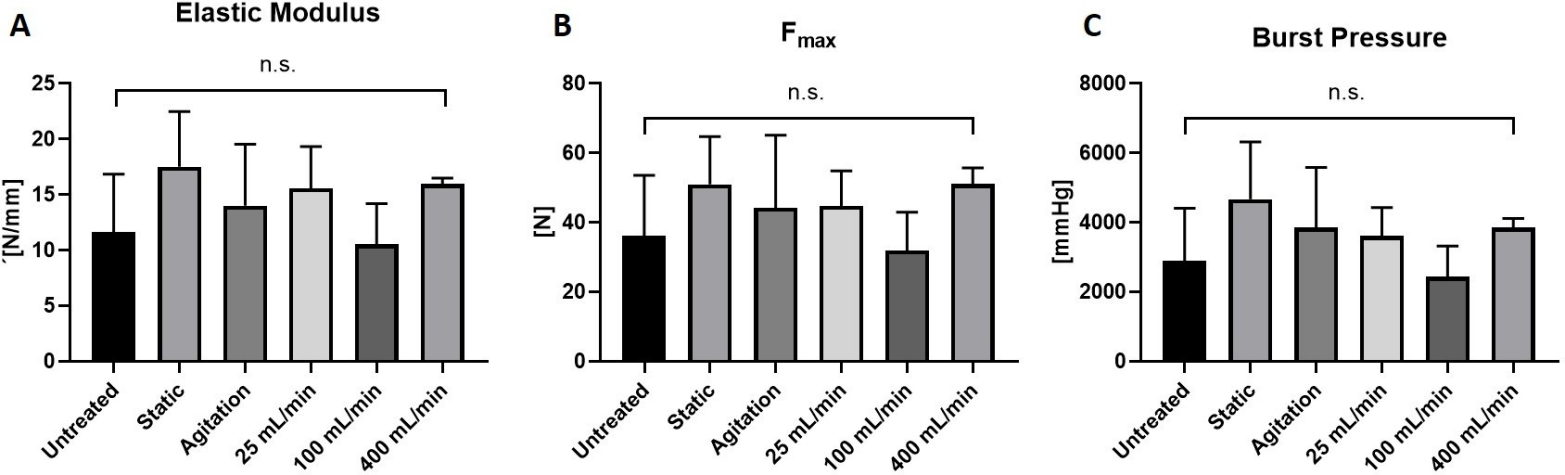
Mechanical testing of untreated and decellularized porcine vena cava. Mechanical testing was performed on equally sized vessel ringlets placed on metal U-holders in a Zwick Roell testing machine. The vessels ringlets were stretched until failure and stress-strain curves were recorded. n = 5 vessels for all groups, 3 ringlets per vessel tested as technical replicates. (A) Elastic Modulus (Youngs Modulus) calculated from linear phase of stress strain curve. Higher values indicates increased stiffness and lower values indicates increased elasticity. (B) F_max_ values (maximum tensile strength). (C) Theoretical burst pressure calculated from vessel properties and F_max_ with Barlows equation.

Mechanical properties of the decellularized veins were analyzed using a ringlet test, in which vein ringlets from each DC group were stretched until failure while stress-strain curves were acquired. Vessel wall thickness was significantly reduced in all decellularized groups compared with untreated samples (p<0.0001). The vessel wall thinning was most pronounced in the perfusion groups (Table 1). Interestingly, no difference between DC and untreated group was found in terms of elastic modulus (Fig 7A), maximum tensile strength F_max_ (Fig 7B) or burst pressure (Fig 7C). Taken together, the DC process caused a thinning of the vessel wall that was most pronounced in the perfusion groups, however, the thinning did not alter mechanical properties.

**Table 1:** Vessel wall thickness. The thickness of untreated and decellularized vena cavas was measured with digital caliper (n = 5, middle column). Results were standardized to the untreated sample (100%) to show reduction of thickness (right column).

	Thickness [mm]	Thickness Standardized to untreated [%]
Untreated	1.17 ± 0.06	100
Static	0.63 ± 0.18	53.71
Agitation	0.55 ± 0.23	47.17
25 mL/min	0.41 ± 0.02	34.95
100 mL/min	0.42 ± 0.04	35.81
400 mL/min	0.39 ± 0.02	33.25

## Discussion

Decellularized blood vessels are promising biomaterials for tissue engineering of vascular grafts due to their native character and structure. However, the different steps in the DC procedure may affect the properties of the decellularized vessels and thus have negative impact for *in vivo* functionality. In this study, we investigated how application of detergents during the DC process (static, agitation and perfusion at 25, 100 or 400 mL/min) influences the vessel properties. We show that DC efficacy, ECM integrity, cytotoxicity, and mechanical properties were comparable between the DC groups. However, SEM analysis revealed ruptures of the luminal surface at high perfusion velocity (400 mL/min). SEM also detected remnants of cells/proteins on the luminal surface of vessels in the static group. Taken together, our results show that multiple methods can achieve efficient decellularization of porcine vena cava with remained mechanical properties. However, the most successful methods to achieve DC vessels free from luminal debris and luminal ruptures are agitation DC and perfusion DC at 25 mL/min or 100 mL/min.

A variety of DC protocols have been established that use different DC reagents and different reagent application methods. Previously, we showed that DC protocols utilizing a combination of the detergents TX and TnBP are preferable when applied on blood vessels, due to a favorable recellularization *in vitro* [11]. The method how DC reagents are applied to the tissue has also been shown to be a critical factor for cell removal and mechanical properties, since fluid dynamics influence the surface contact and physical force of the reagents to the tissue [8]. However, for blood vessels our study shows that efficient decellularization is achieved efficiently in all groups and independent of the detergent application method (static, agitation, and perfusion).

In the present study, we found an overall decrease of vessel wall thickness in all DC groups. This is likely due to a combination of cell removal and loss of ECM components, such as GAGs and soluble collagen. The decrease of vessel wall thickness appeared at a greater extend in the perfused groups, which indicates an effect of the physical force on the protein abundancy. GAGs are the polysaccharide component of proteoglycans in the ECM, and they influence vascular graft function by retaining growth factors, by having mechanosensory properties, and by retaining water [7,25,26]. GAGs are also abundant on cell membranes and part of the GAG loss may thus be related to cell removal in the DC process. Fibronectin and vitronectin are extracellular proteins important for cell-ECM interactions through integrins such as α5β1 [27] or αvβ3[28]. Especially fibronectin has been reported to improve recellularization of vascular grafts [29–31]. Both proteins showed to be abundant in all DC groups at tunica intima and media, with no observable differences in fluorescence intensity.

Soluble collagen is a collagen fraction that is susceptible to acid-pepsin degradation. Similar to our results, soluble collagen has previously been reported to be affected by the DC process as a result of EDTA treatment [32,33]. In contrast, insoluble collagen was present at much larger quantities than soluble collagen, and the content of insoluble collagen was not reduced in any DC group. This is in line with a previous study showing that covalently cross-linked insoluble collagen is not affected by DC treatment [34]. We also found that the elastin content was unchanged by the DC process. Elastin and collagen contribute to the mechanical stability of the ECM [35–37], and indeed the mechanical properties (stiffness, maximum tensile strength, burst pressure) of all DC groups were comparable to the untreated native group. Mechanical properties that resemble native vessels are important for the compliance of vascular grafts. Rigid synthetic grafts, such as Dacron or PTFE, causes compliance mismatches at the anastomoses, which results in pathological flow disturbances and formation of stenotic lesions [38]. Hence, the preserved mechanical properties of DC vessels are advantageous when used as graft conduits.

Although histochemical analyzes showed no major ECM alterations, SEM revealed a few morphological changes at the luminal surface, with luminal raptures and surface flattening in the high perfusion group and remnants of cell/protein debris in the static group. The ruptures at high perfusion are likely a direct result of high shear forces of the detergents acting on the luminal surface, and have been observed also by others [39]. The remnants on the luminal surface in the static group suggest a non-complete removal of cell debris. However, we cannot conclude whether the debris will have negative impact on the graft function *in vivo*. Furthermore, the cytotoxicity assay indicates an efficient washout of detergents in all DC groups, yet these results do not allow conclusions about the suitability of the graft surface to be re-endothelialized, which has to be further investigated.

## Conclusion

In this study, we show that the application mode of the decellularization agents TX, TnBP and DNase does not influence the decellularization efficacy or mechanical properties when applied on porcine vena cava. However, perfusion causes a pronounced thinning of the vessel wall and the highest perfusion velocity (400 mL/min) causes tearing of the luminal surface, while static conditions resulted in cellular remnants on the luminal surface. We conclude that decellularization of blood vessels with agitation or at low perfusion velocities (25 and 100 mL/min) is preferable to static or high velocity perfusion (400 mL/min). Decellularization approaches have great potential in regenerative medicine for multiple organ types. This study underscores that blood vessels are a relatively simple organ to decellularize, but care must be taken to avoid undesirable alterations of the luminal surface. Understanding how the choice decellularization method affects the properties of blood vessels is of major importance for the clinical translation of this technology.
